# No Quack Advertisements

**Published:** 1899-12

**Authors:** 


### No Quack Advertisements.

There is at least one daily religious newspaper in the world. It is the Montreal *Witness*, of Canada. It has 200,000 readers and is edited by the son of a sturdy Scotchman, John R. Dougall. It sacrifices about $50,000 a year because it refuses to advertise quack medicines, and theater and sporting gossip. To its wholesome influence is ascribed the fact that none of the Canadian dailies issue Sunday editions.

